# Mesenchymal Stem Cells‐Involved Strategies for Rheumatoid Arthritis Therapy

**DOI:** 10.1002/advs.202305116

**Published:** 2024-03-13

**Authors:** Chaoyang Li, Yifu Sun, Weiguo Xu, Fei Chang, Yinan Wang, Jianxun Ding

**Affiliations:** ^1^ Department of Orthopedics The Second Hospital of Jilin University 4026 Yatai Street Changchun 130041 P. R. China; ^2^ Key Laboratory of Polymer Ecomaterials Changchun Institute of Applied Chemistry Chinese Academy of Sciences 5625 Renmin Street Changchun 130022 P. R. China; ^3^ Department of Biobank Division of Clinical Research The First Hospital of Jilin University 1 Xinmin Street Changchun 130061 P. R. China; ^4^ Key Laboratory of Organ Regeneration and Transplantation of the Ministry of Education The First Hospital of Jilin University 1 Xinmin Street Changchun 130061 P. R. China

**Keywords:** biomaterial, inflammation inhibition, mesenchymal stem cell, rheumatoid arthritis therapy, tissue regeneration

## Abstract

Rheumatoid arthritis (RA) is a systemic autoimmune disease characterized by chronic inflammation of the joints and bone destruction. Because of systemic administration and poor targeting, traditional anti‐rheumatic drugs have unsatisfactory treatment efficacy and strong side effects, including myelosuppression, liver or kidney function damage, and malignant tumors. Consequently, mesenchymal stem cells (MSCs)‐involved therapy is proposed for RA therapy as a benefit of their immunosuppressive and tissue‐repairing effects. This review summarizes the progress of MSCs‐involved RA therapy through suppressing inflammation and promoting tissue regeneration and predicts their potential clinical application.

## Graphical abstract

Mesenchymal stem cells reduce the secretion of inflammatory cytokines and inhibit synovial inflammation by acting on immune cells, such as macrophages, T cells, and B cells, and differentiate into osteoblasts and chondroblasts and promote tissue repair, providing a promising approach to treat rheumatoid arthritis.

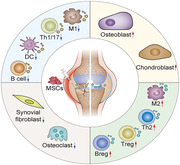

## 1 Introduction

Rheumatoid arthritis (RA) is an autoimmune disease with a prevalence rate of more than 1% worldwide. Most of the patients are middle‐aged and elderly, and the prevalence rate in females is higher than that in males.^[^ ^1^ ^]^ Inflammation of the synovium and joints results in bone destruction and deformities in RA.^[^ ^2^ ^]^ The pathogenesis of RA involves the activation of pro‐inflammatory cytokines and the immune system. Various immune cells produce many pro‐inflammatory cytokines, leading to joint and synovium inflammation.^[^ ^3^ ^]^

At present, the commonly used drugs for RA therapy are glucocorticoids (GCs), non‐steroidal anti‐inflammatory drugs (NSAIDs), disease‐modifying anti‐rheumatic drugs (DMARDs), and biological agents like tumor necrosis factor (TNF) inhibitors.^[^ ^4^ ^]^ However, traditional drugs for RA usually relieve symptoms only by reducing pain and inflammation and cannot eliminate the cause—so the effect of these drugs is not ideal. Less than half of RA patients are in remission, and 10%–15% develop refractory RA.^[^ ^5^ ^]^ In addition, the lack of specificity of traditional drugs for RA and the need for high doses will lead to a series of side effects. For example, high doses of GCs may lead to Cushing's syndrome, high blood pressure, and diabetes.^[^ ^6^ ^]^ In addition, methotrexate (MTX) is associated with the liver and kidney injury, pulmonary fibrosis, and cancer risk.^[^ ^7^ ^]^ Furthermore, TNF inhibitors may cause infection, lymphoma, and neurological disorders in patients.^[^ ^8^ ^]^

**Scheme 1:**
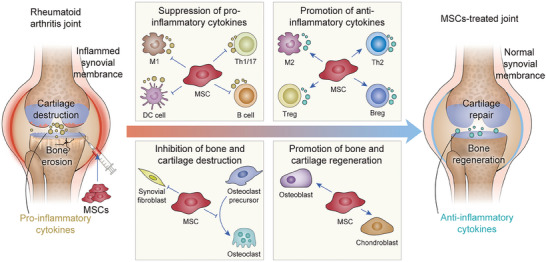
MSCs‐involved strategies for RA therapy.

Given the shortcomings of traditional therapeutic drugs, there is a growing focus on mesenchymal stem cells (MSCs) due to their immunomodulatory properties and tissue regeneration ability. MSCs have low immunogenicity and homing characteristics and directionally migrate to injured tissues or inflammatory sites. As a multi‐tissue‐derived cell, MSCs can be easily isolated from many tissues.^[^ ^9^ ^]^ The pluripotent differentiation properties of MSCs have led to their widespread use in regenerative medicine.^[^ ^10^ ^]^ Besides, MSCs regulate immunity and inhibit inflammation by direct contact, secreting soluble factors or extracellular vesicles.^[^ ^11^ ^]^ Therefore, when used for RA therapy, MSCs regulate immunity, inhibit inflammation, and differentiate into osteoblasts and chondrocytes to repair damaged articular tissues (**Scheme** **1**).^[^ ^12^ ^]^ This review discusses the application of MSCs in RA therapy by inhibiting inflammation and promoting tissue repair, as shown in **Table** **1**, and prospects for their potential clinical application future.

**Table 1:** Summary of MSCs‐involved Strategies for RA therapy.

Mechanism of Action	Source of MSCs	Methods to enhance MSCs	Therapeutic outcome	Type of RA model	Reference
Suppression of Pro‐Inflammatory Cytokines	BM‐MSCs	Microfracture and orthotopic transplantation of thermal gel‐encapsulated MSCs	Reduced inflammatory cytokine levels and inhibited joint inflammation	CIA	[33]
	BM‐MSCs	IFN‐γ receptor knockout MSCs and wild‐type MSCs	Only wild‐type MSCs significantly improved joint inflammation	CIA	[39]
	UC‐MSCs	TNFR2‐MSCs expressing TNF‐α2	Blocked TNF‐α to reduce joint inflammation and cartilage destruction	CIA	[42]
	BM‐MSCs	Micro‐circular vectors encoding biopharmaceutical sequences to synthesize mcTNFR2‐MSCs	Anti‐inflammatory and anti‐osteoclastogenic effects	CIA	[43]
	BM‐MSCs	Encapsulated MSCs in alginate hydrogel	Inhibited DCs and reduced inflammation	CIA	[46]
Promotion of Anti‐Inflammatory Cytokines	A‐MSCs	G3K/OHA hydrogel loaded with A‐MSCs	Inhibited inflammation and reduced RA symptoms	CIA	[70]
	BM‐MSCs	Combined treatment of MSCs and HSD	Improved the inflammatory cell infiltration, synovial hyperplasia	AIA	[75]
	BM‐MSCs	Caffeine‐pulsed MSCs	Inhibited inflammation and reduced disease severity	CIA	[79]
	BM‐MSCs	Combined treatment with MSCs and IL‐4	Reduced joint inflammation and synovial cellularity	CIA	[84]
	UC‐MSCs	MSCs nanovesicle system containing ceria	Improved symptoms of inflammation and arthritis	CIA	[86]
	A‐MSCs	A‐MSCs with over‐expression of CTLA4Ig	Improved immunomodulation and symptoms	CIA	[89]
	BM‐MSCs	MSCs over‐expressing CXCR7	Inhibited inflammation and ameliorated arthritic symptoms	CIA	[90]
	BM‐MSCs	BM‐MSCs with over‐expression of IL‐10	Inhibited inflammation and increased joint cartilage repair	CIA	[92]
	A‐MSCs	MiR‐146a transduced MSC‐EVs	Reproduced the immunological potential of MSCs and maintained the immune balance	CIA	[93]
Inhibition of Bone and Cartilage Destruction	A‐MSCs	DS‐EXOs derived from metabolic engineering editing of A‐MSC	Promoted M1 to M2 polarization and decreased arthritis score	CIA	[111]
	BM‐MSCs	Fibrin gel and hydrogel‐loaded MSCs	Reduced FLS proliferation and cartilage destruction	AIA	[123]
	BM‐MSCs	MSC‐derived exosomes loaded with microRNA‐320a	Reduced bone and joint injury and arthritis progression	CIA	[127]
	BM‐MSCs	Loaded GVs into MSCs and combined with MTX	Decreased arthritis index score and bone erosion and cartilage destruction	CIA	[130]
Promotion of Bone and Cartilage Regeneration	A‐MSCs	A‐MSCs loaded with 3D metal scaffold loaded and infliximab hydrogel	Reduced cartilage damage and improved repair effect	CIA	[142]
	BM‐MSCs	MSCs modified by VQ‐CuS@MnO_2_/MET	Enhanced chondrogenesis and synovial reduced inflammation	CIA and AIA	[143]
	BM‐MSCs	Nano‐enzyme enhanced hydrogel as a carrier of MSCs	Enhanced the effect of bone regeneration and bone integration	OVA‐induced RA rabbit	[144]
	BM‐MSCs	Implanted sIL‐6R‐pretreated MSCs onto poly membranes	Enhanced articular cartilage repair	AIA	[151]

## 2 Inhibition of Inflammation

RA is an autoimmune disease whose pathogenesis involves innate immunity, acquired immunity, cytokines, and intracellular signaling.^[^ ^13^ ^]^ The anti‐inflammatory effect of MSCs is achieved by interacting with immune cells. Intercellular contact, paracrine effects, and extracellular vesicles mediate immunomodulatory processes.^[^ ^14^ ^]^ MSCs produce a series of soluble factors involved in the immunomodulatory axis, such as transforming growth factor‐β1 (TGF‐β1), prostaglandin E‐2 (PGE‐2), hepatocyte growth factor (HGF), indoleamine‐pyrrole 2, 3‐dioxygenase (IDO), nitric oxide (NO), and interleukin‐10 (IL‐10).^[^ ^15^ ^]^

Although it is generally believed that soluble factors mainly achieve the immunomodulatory function of MSCs, MSCs‐derived extracellular vesicles (MSC‐EVs) also mediate immunomodulatory responses. EVs are nano‐vesicles coated with phospholipid bilayers, which transfer bioactive molecules from parent cells to receptor cells, such as proteins, lipids, and nucleotides.^[^ ^16^ ^]^ MSC‐EVs are endosome‐derived vesicles and are 100–200 nm in size. MSC‐EVs express neither major histocompatibility complex molecules nor costimulatory molecules.^[^ ^17^ ^]^ Moreover, MSC‐EVs inhibit the development of activated T cells while promoting regulatory T cell (Treg) production.^[^ ^18^ ^]^ Moreover, MSC‐EVs inhibit B cell proliferation, differentiation, and immunoglobulin production.^[^ ^19^ ^]^ In innate immunity, MSC‐EVs promote M2 macrophage polarization, reduce pro‐inflammatory cytokines, and enhance anti‐inflammatory cytokines.^[^ ^20^ ^]^

### 2.1 Suppression of Pro‐Inflammatory Cytokines

RA is pathogenesis‐dependent on pro‐inflammatory cytokines. The imbalance of pro‐inflammatory and anti‐inflammatory cytokines may lead to multi‐system immune complications. Helper T (Th) cells like Th1, Th17, Th22, and regulatory T cells (Tregs), regulate chronic inflammation in RA.^[^ ^21^ ^]^ In addition, the imbalance of the Th17/Treg ratio is also a characteristic of RA.^[^ ^22^ ^]^

The immunomodulatory effect of MSCs is realized mainly by inhibiting effector T cell activation and proliferation by secreting various soluble factors.^[^ ^23^ ^]^ For example, TGF‐β and IL‐10 secreted by MSCs inhibit T cell activity, thereby regulating immune homeostasis.^[^ ^9^ ^]^ Interferon‐γ (IFN‐γ)‐mediated inhibition of T cell proliferation by MSCs may be achieved through the up‐regulation of PGE‐2, TGF‐β1, and HGF.^[^ ^24^ ^]^ MSCs partially activate Tregs through PGE‐2 and convert the more destructive Th1 cells into Th2 and Th17 cells with less destruction of tissues.^[^ ^25^ ^]^ MSCs express IDO when induced by IFN‐γ, which inhibits effector T cell activation.^[^ ^27^ ^]^ When cocultured with Treg‐rich lymphocytes, MSCs also maintained the proportion of Treg cells, thus effectively maintaining immune tolerance.^[^ ^26^ ^]^ In another study, MSCs from RA patients and healthy people were cocultured with peripheral blood mononuclear cells (PBMC). After coculture, inflammatory cytokines decreased while anti‐inflammatory cytokines increased.^[^ ^27^ ^]^ Vasilev et al. cocultured PBMC from 17 RA patients with a conditioned medium of adipose MSCs. After coculture, TGF‐β1 and forkhead box P3 (FoxP3) levels were significantly increased, suggesting that Th17 decreased and Treg increased.^[^ ^28^ ^]^ The result indicates that MSCs inhibit the immune function of lymphocytes in vitro.

The ability of MSCs to regulate immunity and improve the symptoms of RA by regulating T cells in vivo has also been proved. Tregs in the spleen and peripheral blood of collagen‐induced arthritis (CIA) mice treated with human adipose‐derived mesenchymal stem cells (A‐MSCs) increased significantly, suggesting that immune tolerance in mice was enhanced.^[^ ^29^ ^]^ Th1, Treg, and endogenous IDO were increased in the CIA mice treated with embryonic MSCs, while Th17 showed no improvement.^[^ ^30^ ^]^ Repeated intravenous injection of human umbilical cord‐derived mesenchymal stem cells (UC‐MSCs) for a long time in the CIA mouse model increased Treg's content. In the CIA mice, MSCs alleviated arthritis symptoms.^[^ ^31^ ^]^ In addition, UC‐MSCs reduced T follicular helper cells (Tfh) number by secreting IDO, alleviating disease progression in the CIA mice. Notably, MSCs also inhibited the differentiation of Tfh into Tfh1, Tfh2, and Tfh17, reducing the production of autoreactive antibodies.^[^ ^32^ ^]^ In the CIA model, treatment with microfracture and thermal gel‐encapsulated MSCs orthotopic transplantation decreased the cluster of differentiation 4^+^ (CD4^+^)/CD8^+^ T cell ratio. Besides, antigen‐specific lymphocytes and inflammatory cytokines levels in the serum were significantly decreased.^[^ ^33^ ^]^

Although MSCs improve the symptoms of RA by regulating the activity and proliferation of T cells, the regulatory capacity is limited in the inflammatory microenvironments of RA. Interestingly, appropriate inflammatory factors are necessary to exert the immunomodulatory ability of MSCs. They play a “permission” role in the immune regulation process by MSCs.^[^ ^34^ ^]^ Moreover, inflammatory signaling is necessary for MSCs to exert their immunosuppressive effect. Without appropriate inflammatory stimulation, the immunosuppressive effects of MSCs are difficult to manifest. For example, A‐MSCs have been shown to modulate immunity in the inflammatory synovial fluid but not in normal synovial fluid due to the lack of an appropriate inflammatory microenvironment.^[^ ^35^ ^]^ Bone marrow‐derived mesenchymal stem cells (BM‐MSCs) were cultured in the conditioned medium containing synovial fluid from RA patients. IDO and IL‐6 expression in MSCs was higher than that in the medium without synovial fluid. In addition, the inhibitory effect of MSCs conditioned medium added to synovial fluid on lymphocyte proliferation was significantly enhanced.^[^ ^36^ ^]^ Therefore, improving the regulatory impact of MSCs on T cells is feasible by regulating an appropriate inflammatory microenvironment.

To regulate the inflammatory microenvironments and enhance the immune regulatory effect of MSCs on T lymphocytes, the researchers focused on inflammatory cytokines, such as IFN and TNF. The pretreatment of MSCs with IFN‐γ enhances the regulatory effect on T cells, improving their immunosuppressive effect. IFN‐γ is a major inflammatory cytokine in the pathogenesis of RA, mainly secreted by CD8^+^ T cells and abundantly present in the RA joints. The pretreatment of MSCs with IFN‐γ increased the expression of inhibitory factors. These inhibitors down‐regulate T cell activation, increase negative T cell signaling, and increase the Treg/Th17 ratio.^[^ ^37^ ^]^ Human dental follicle‐derived MSCs co‐incubated with IFN‐γ in vitro were shown to enhance Treg increase in RA patients further, increase T cell activity in RA patients, and inhibit T cell apoptosis.^[^ ^38^ ^]^ He et al. investigated the therapeutic effects of IFN‐γ receptor knockout MSCs and wild‐type MSCs on CIA mice. The results showed that only wild‐type MSCs significantly improved the joint inflammation in the CIA mice.^[^ ^39^ ^]^ This further verifies the enhanced effect of IFN‐γ pretreatment on the T‐cell regulatory capacity of MSCs in vivo.

**Figure 1:**
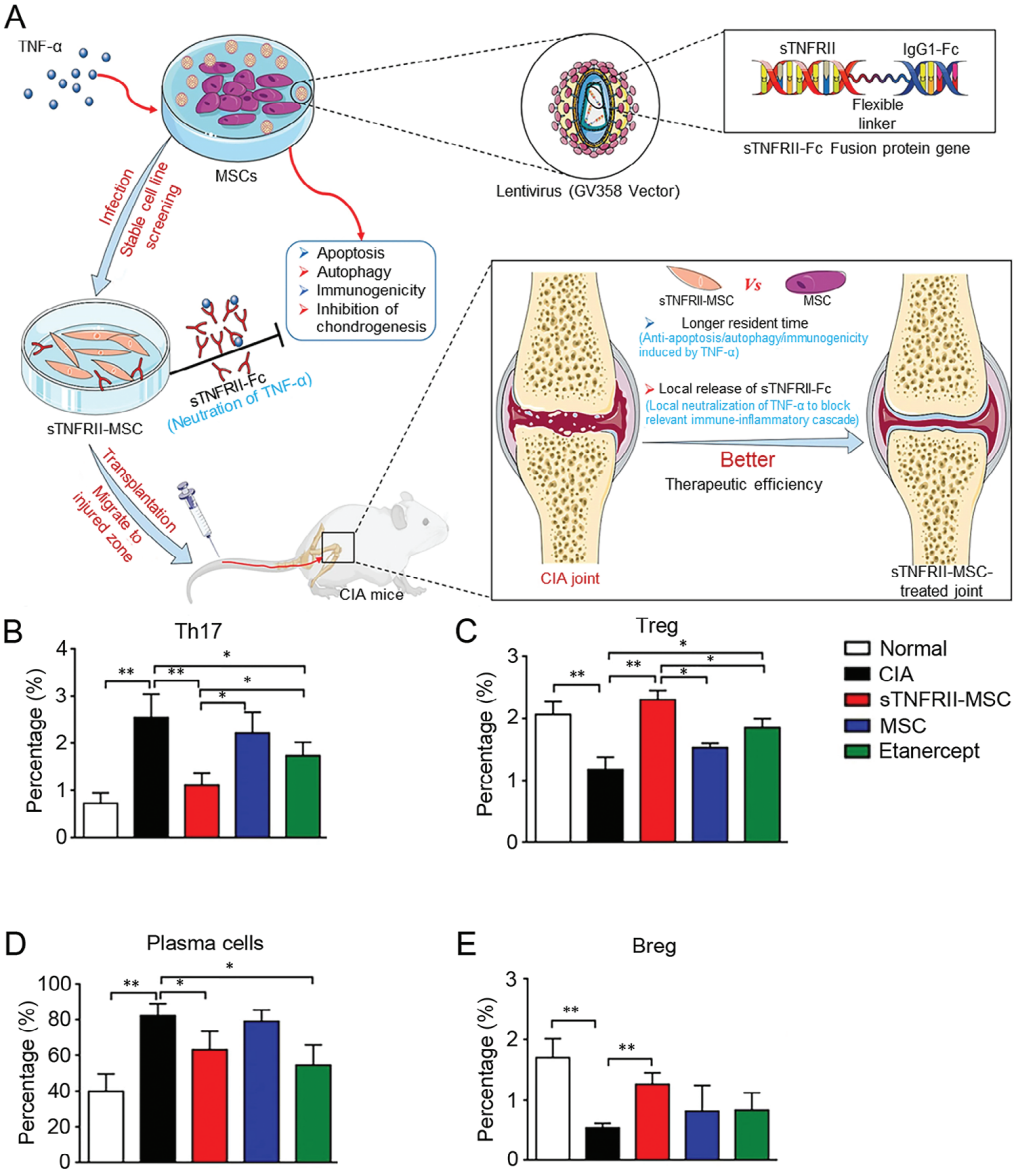
Mechanism and effect of TNFRII‐MSCs in treatment of CIA mice. A) Mechanism of TNFRII‐MSCs in CIA therapy. B,C) TNFRII‐MSCs regulate Th17 and Treg in spleens of CIA mice. ^***^ *p* < 0.001). D,E) TNFRII‐MSCs regulate plasma cells and Breg cells in spleens of CIA mice. Data are shown as mean ± standard deviation (SD; *n* = 8; ^*^ *p* < 0.05, ^**^ *p* < 0.01, ^***^ *p* < 0.001). Reproduced under the terms of the CC‐BY license.^[^ ^42^ ^]^ Copyright 2021, the authors.

Although an appropriate inflammatory microenvironment is indispensable for MSCs to exert immunosuppressive effects, an excessive inflammatory microenvironment, especially TNF‐α, reduces the immunosuppressive capacity of MSCs by inducing apoptosis.^[^ ^34^ ^]^ The level of TNF‐α in plasma and synovial fluid increased in the RA microenvironments, reflecting disease activity. Elevated levels of TNF‐α lead to RA pathogenic cell proliferation and increase pro‐inflammatory cytokines, chemokines, and other pathogenic factors, aggravating RA progression.^[^ ^40^ ^]^ Therefore, TNF‐α blockers like Etanercept have been widely used to treat RA. Etanercept is synthesized by soluble TNF‐α2 receptor (TNFR2) and Fc domain of human immunoglobulin, which competitively inhibits TNF‐α.^[^ ^41^ ^]^ To eliminate the adverse effects of TNF‐α on MSCs, Zhao et al. prepared TNFR2‐MSCs expressing TNF‐α2 and injected them into CIA mice (**Figure** **1**A).^[^ ^42^ ^]^ Th17 and plasma cells are pathogenic cells in RA, secreting pro‐inflammatory cytokines and autoantibodies. TNFR2‐MSCs significantly reduced Th17 and plasma cells (Figure 1B,D). Interestingly, TNFR2‐MSCs treatment increased Tregs and regulatory B cells (Bregs) in the spleens of CIA mice. The effect was more substantial than that of wild‐type MSCs (Figure 1C,E). Park et al. used micro‐circular vectors encoding biopharmaceutical sequences to synthesize mcTNFR2‐MSCs. Compared with non‐engineered MSCs, inhibition of Th17 cells by mcTNFR2‐MSCs was more pronounced.^[^ ^43^ ^]^

**Figure 2:**
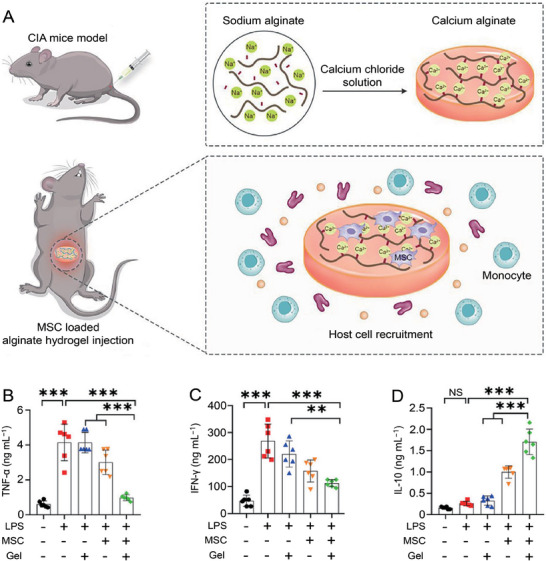
Mechanism and effect of encapsulated MSCs in RA therapy. A) The proposed mechanism of encapsulated MSCs in RA therapy. B) Inhibition of inflammatory mediator TNF‐α in LPS‐stimulated BMDCs treated with encapsulated MSCs. C) Expression of inflammatory mediator IFN‐γ in LPS‐stimulated BMDCs treated with encapsulated MSCs. D) Promotion of anti‐inflammatory mediator IL‐10 in LPS‐stimulated BMDCs treated with encapsulated. Data are shown as mean ± ) P (*n* = 3; ^**^ *p* < 0.01, ^***^ *p* < 0.001). Reproduced with permission.^[^ ^46^ ^]^ Copyright 2023, Elsevier.

In addition to T cells, RA pathogenesis is related to dendritic cells (DCs). DCs are present in inflammatory synovial tissue. DCs act as antigen‐presenting cells that capture, process, and present antigens to T cells, leading to T cell activation and differentiation.^[^ ^44^ ^]^ In addition, DCs secrete pro‐inflammatory cytokines, promoting chronic inflammation of the joints in RA patients.^[^ ^45^ ^]^ When used in RA therapy, the antigen presentation function of DC is inhibited by MSCs. Therefore, regulating DCs for RA therapy is a potential method. By regulating DC function, MSCs inhibit immune response and reduce RA inflammatory response. Shi et al. encapsulated MSCs in alginate hydrogel for CIA treatment (**Figure** **2**A).^[^ ^46^ ^]^ After treatment, tolerance DCs in the CIA mice increased, resulting in a significant increase of Treg. Encapsulated MSCs significantly decreased TNF‐α and TFN‐ γ expression in DCs in vitro (Figure 2B,C). In addition, the anti‐inflammatory cytokine IL‐10 was significantly increased (Figure 2D). MSCs coated with alginate hydrogel alleviated arthritis in the CIA mice by regulating DCs.

The ability of MSCs to inhibit pro‐inflammatory cytokines is realized by inhibiting immune cells like M1 macrophages, Th1, DCs, and B cells. Appropriate inflammatory stimulation is conducive to the immunomodulatory function of MSCs, while excessive inflammatory stimulation inhibits the immunomodulatory function of MSCs. The following questions that must be explored are how to reduce the effect of RA inflammatory microenvironments on MSCs and maintain the anti‐inflammatory ability of MSCs.

### 2.2 Promotion of Anti‐Inflammatory Cytokines

Anti‐inflammatory cytokines have biological effects of inhibiting inflammatory response and immune response. In RA, the production of anti‐inflammatory cytokines is usually regulated and mediated by immune cells, including T cells, B cells, and M2 macrophages. Specific T cell subsets like Th2 and Tregs secrete anti‐inflammatory cytokines. B cells are another essential effector cells in RA.

MSCs exert an inhibitory effect on B cells by inhibiting B cell proliferation and differentiation.^[^ ^47^ ^]^ Conditioned culture medium produced from human placental amniotic MSCs inhibits effector B cell formation and differentiation.^[^ ^48^ ^]^ BM‐MSCs reduced plasma cell generation, which may be caused by the decreased expression of miRNAs required for B cell maturation through humoral factors secreted by BM‐MSCs.^[^ ^49^ ^]^ In addition, the coculture of BM‐MSCs with B cells down‐regulated immunoglobulins and chemokine receptors in B cells.^[^ ^3^ ^]^ The effect of MSCs is mainly through the secretion of soluble cytokines. For example, IL‐1 receptor antagonists from MSCs inhibit B cell differentiation and joint inflammation progression.^[^ ^50^ ^]^ C‐C chemokine ligand 2(CCL2) produced by MSCs inhibits transcriptional activator (SATA3) production in plasma cells, inhibiting immunoglobulin synthesis after treatment with metalloproteinase.^[^ ^51^ ^]^ In addition to soluble cytokines, MSC‐EVs also inhibit the proliferation, differentiation, and antibody production of B cells in a dose‐dependent manner.^[^ ^52^ ^]^ MSCs induce Breg production to mediate anti‐inflammatory effects. Bregs secrete anti‐inflammatory cytokines like IL‐10 to inhibit the immune response.^[^ ^53^ ^]^

**Figure 3:**
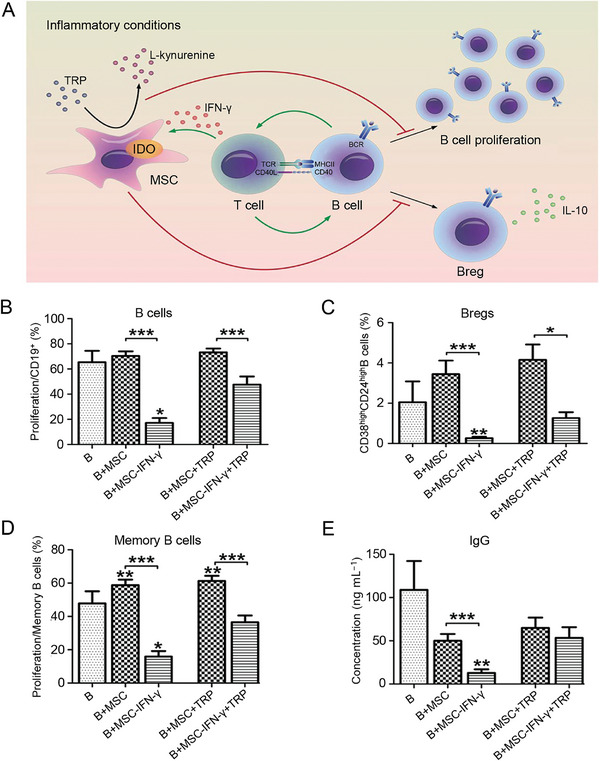
Mechanism and effect of MSCs on B cells in an inflammatory microenvironment. A) Interconnection between MSCs and B cells under inflammatory conditions. B–D) Proportion of each cell line after coculture of B cells and MSC for seven days. 1, ^***^ *p* < 0.001). E) Content of IgG in culture supernatant. Data are shown as mean ± SD (*n* = 3; ^*^ *p* < 0.05, ^**^ *p* < 0.01, ^***^ *p* < 0.001). Reproduced under the terms of the CC‐BY license.^[^ ^57^ ^]^ Copyright 2017, the authors.

Like T cells, the inhibition of B cells also requires appropriate inflammatory stimulation to initiate. For example, appropriate IFN‐γ stimulation is indispensable for the immunosuppressive function of MSCs.^[^ ^54^ ^]^ Sufficient inflammatory signals, such as mycoplasma arginine, enhanced B cell antibody secretion inhibition by MSCs.^[^ ^55^ ^]^ When the inflammatory signal stimulation is insufficient, the inhibitory ability of MSCs to B cells is significantly reduced and may even increase the antibody secretion of B cells.^[^ ^56^ ^]^ Under immune quiescence, the MSCs‐induced Breg production but did not affect B cell proliferation and immunoglobulin secretion. MSCs pretreated with IFN‐γ inhibited B cell proliferation, immunoglobulin production, and Breg induction (**Figure** **3**A).^[^ ^57^ ^]^ This inhibition may be due to the suppression of all B cells and may depend on IDO‐mediated tryptophan depletion. Adding tryptophan to the coculture system reversed the inhibitory effect of MSC on IgG and memory B cells (Figure 3B,C). In addition, tryptophan supplementation partially reversed the promoting effect of MSC on Breg and IL‐10 (Figure 3D,E).

A study on mesenchymal stem cells from pediatric patients (PMSCs) also found that PMSCs stimulated by IFN‐γ significantly inhibited the proliferation of B cells in all subsets, while PMSCs without IFN‐γ stimulation had no such effect.^[^ ^58^ ^]^ In conclusion, appropriate inflammatory factors enhance the regulation of B cells. By contrast, a lack of inflammatory signals or excessive inflammatory stimuli is detrimental to the regulation ability of MSCs.

Although the inflamed joints of RA are filled with various inflammatory cells, macrophages are the major players.^[^ ^59^ ^]^ It has been found that innate immunity is essential in RA pathogenesis. Innate immune cells activate the adaptive immune system, which plays a crucial role in the later stages of RA.^[^ ^60^ ^]^ Innate immune cells produce cytokines and chemokines, leading to the continuous entry of immune cells and participation in subsequent immune processes.^[^ ^61^ ^]^ Macrophages are heterogeneous and can be polarized into M1 or M2 types.^[^ ^62^ ^]^ M1‐type macrophages are pro‐inflammatory cells and secrete various pro‐inflammatory cytokines in the RA‐inflamed joints.^[^ ^63^ ^]^ By contrast, M2‐type macrophages produce anti‐inflammatory cytokines with anti‐inflammatory and tissue repair properties.^[^ ^64^ ^]^ A balance of pro‐ and anti‐inflammatory cytokines is critical in RA progression. In RA patients, M1 macrophages secret more TNF‐α and IL‐1, while M2 macrophages secret less IL‐10 than those in healthy people.^[^ ^65^ ^]^ Macrophages also produce reactive oxygen species (ROS), including C‐X‐C chemokine ligand 8 (CXCL8) and CCL2, to promote the progression of RA. These factors are critical for recruiting neutrophils and monocytes to inflamed joint sites.^[^ ^66^ ^]^

MSCs regulate macrophage polarization to maintain the balance of inflammatory cytokines. When cocultured with macrophages, MSCs promote M2‐type macrophage polarization by secreting soluble factors, such as IDO, PGE‐2, IL‐10, and cyclooxygenase‐2. Through the participation of soluble factors, MSCs enhance the anti‐inflammatory phenotype of macrophages and inhibit their pro‐inflammatory phenotype.^[^ ^67^ ^]^ IL‐1 receptor antagonist (IL‐1Ra) is critical in the MSCs‐mediated macrophage polarization.^[^ ^50^ ^]^ In addition, MSCs also promote IL‐10 secretion and inhibit IL‐12 and TNF‐α secretion in macrophages.^[^ ^68^ ^]^ The above effects of MSCs on macrophages have been verified in RA. In the CIA model, IL‐1Ra knockout MSCs to induce M2 macrophage polarization was significantly reduced compared to normal MSCs, and the effect of improving joint inflammation was completely attenuated.^[^ ^50^ ^]^

**Figure 4:**
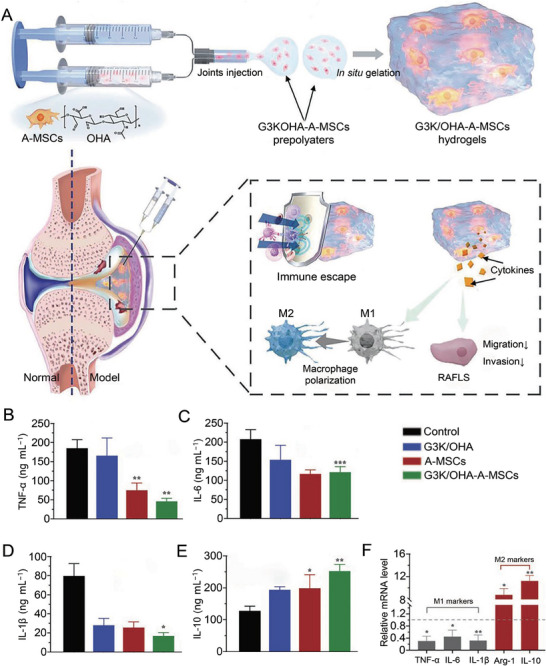
Mechanism and effect of A‐MSCs‐laden bioinspired hydrogel in RA therapy. A) The proposed mechanism of G3K/OHA‐A‐MSCs hydrogel in RA therapy. B–E) Supernatant cytokines in BDMDs treated by G3K/OHA, A‐MSCs, and G3K/OHA‐A‐MSCs. F) mRNA levels of M1 macrophage markers (IL‐1β, IL‐6, and TNF‐α), and M2 macrophage markers (Arg‐1 and IL‐10) in activated macrophages without and with treatment of G3K/OHA‐A‐MSCs hydrogels. Data are shown as mean ± SD (*n* = 3; ^*^0.01 < *p* < 0.05, ^**^0.001 < *P* < 0.01, ^***^ *p* < 0.001). Reproduced under the terms of the CC‐BY license.^[^ ^70^ ^]^ Copyright 2023, the authors.

Intravenous injection of human umbilical cord MSCs after the onset of CIA mice significantly improved the local and systemic inflammatory responses. Coculture of human umbilical cord MSCs with PBMCs of RA patients further verified the anti‐inflammatory effect of MSCs.^[^ ^69^ ^]^ However, the intravenous administration of MSCs may result in insufficient cell distribution and decreased viability at the inflammatory joints. As an alternative to intravenous injection, in situ injection of MSCs into the inflamed joints with hydrogel is a promising approach. Zhu et al. developed an injectable hydrogel loaded with A‐MSCs to treat RA. The hydrogel is composed of polylysine dendrimer (G3K) and oxidized hyaluronic acid (OHA) (**Figure** **4**A).^[^ ^70^ ^]^ After in situ injection into the joint cavity, the hydrogel provides a suitable microenvironment for A‐MSCs and increases the anti‐inflammatory ability of A‐MSCs. The hydrogel‐loaded A‐MSCs significantly inhibited the production of TNF‐α, IL‐6, and IL‐1β (Figure 4B–D). In addition, the secretion of the anti‐inflammatory cytokine IL‐10 increased significantly (Figure 4E). Macrophage markers before and after hydrogel‐A‐MSCs treatment demonstrated that this anti‐inflammatory ability is mainly generated by regulating macrophage phenotype from M1 to M2 (Figure 4F). Besides, MSCs‐derived exosomes induce M2 polarization in monocytes and secrete IL‐10 and TGF‐β.^[^ ^71^ ^]^

Macrophages are the central pro‐inflammatory cells in the RA‐inflamed joints that produce inflammatory cytokines, ROS, NO intermediates, and other factors.^[^ ^66^ ^]^ The oxidative stress microenvironments and excessive ROS production at RA patients' inflamed joint sites significantly reduced the survival rate of MSCs after transplantation and affected the efficacy.^[^ ^72^ ^]^ ROS in the RA joints is mainly derived from macrophages and neutrophils in the synovial cavity. Antioxidants neutralize free radicals and inhibit the oxidative reaction chain on mitochondria to reduce ROS production, thereby protecting the cellular components.^[^ ^73^ ^]^

Interestingly, the combined use of antioxidants in stem cell therapy neutralized the oxidative microenvironments and improved the anti‐stress ability of stem cells. In addition, there were improvements in the survival rate of MSCs as well as the immunomodulatory ability and anti‐inflammatory effect of stem cells.^[^ ^72^ ^]^ Hesperidin (HSD) is a flavonoid with anti‐inflammatory, antioxidant, and anti‐rheumatic effects.^[^ ^74^ ^]^ The combined treatment of MSCs and HSD in the antigen‐induced arthritis (AIA) rats significantly reduced inflammatory cytokines like IFN‐γ and up‐regulated TGF‐β levels compared with the treatment of MSCs alone.^[^ ^75^ ^]^ This combination enhances the immunomodulatory effects of MSCs. Caffeine, a xanthine alkaloid, is a competitive antagonist of adenosine, regulating MSCs proliferation and differentiation. Therefore, caffeine alters the interaction between MSCs and immune cells,^[^ ^76^ ^]^ and the caffeine‐pretreated MSCs activate anti‐inflammatory immune cells.^[^ ^77^ ^]^ In another in vitro experiment, caffeine‐pulsed MSCs conditioned medium significantly down‐regulated the expression of ROS and NO in macrophages.^[^ ^78^ ^]^ Caffeine‐pulsed MSCs reduced arthritis index, serum IL‐1β, NO, and TNF‐α levels in the CIA model, and the effect is more potent than wild MSCs.^[^ ^79^ ^]^ Cervus and Cucumis peptides (LG) are biological factors containing osteoinductive biological polypeptides. LG contains TGF‐β, which assists macrophage‐derived cytokines in tissue repair.^[^ ^80^ ^]^ In addition, it inhibits inflammatory response and reduces TNF‐α levels in the serum.^[^ ^80^, ^81^ ^]^ In vitro, the secretion of anti‐inflammatory factors, such as HGF, PGE‐2, and TNF, was remarkably increased in the LG‐treated MSCs group.^[^ ^82^ ^]^ However, the immunomodulatory effect of LG on MSCs and its mechanism need to be improved. IL‐4 is an anti‐inflammatory cytokine that inhibits pro‐inflammatory cells and enhances anti‐inflammatory effects. IL‐4 has also shown disease‐modifying effects in the RA animal models.^[^ ^83^ ^]^ In the CIA mice, combined treatment with MSCs and IL‐4 reduced pro‐inflammatory cytokines in the joints and reduced joint inflammation. Compared with the treatment with MSCs alone, IL‐10 was significantly increased in the combination treatment group, contributing to maintaining the Th1/Th2 balance.^[^ ^84^ ^]^ Ceria, as an antioxidant for scavenging ROS, has been used in RA treatment. Wang et al. prepared gold nanomaterial coated with ceria for local treatment of CIA mice. The nanomaterial effectively inhibited inflammatory cytokines and improved arthritis symptoms. Further, another study combined ceria with MSCs.^[^ ^85^ ^]^ Koo et al. developed a nano‐vesicle system of MSCs containing ceria. The system showed both the antioxidant properties of ceria and the immunomodulatory properties of MSCs. This system promoted Treg cell differentiation and M2 cell polarization in vitro. Besides, the symptoms of inflammation and arthritis in the CIA mice were significantly improved after treatment.^[^ ^86^ ^]^

**Figure 5:**
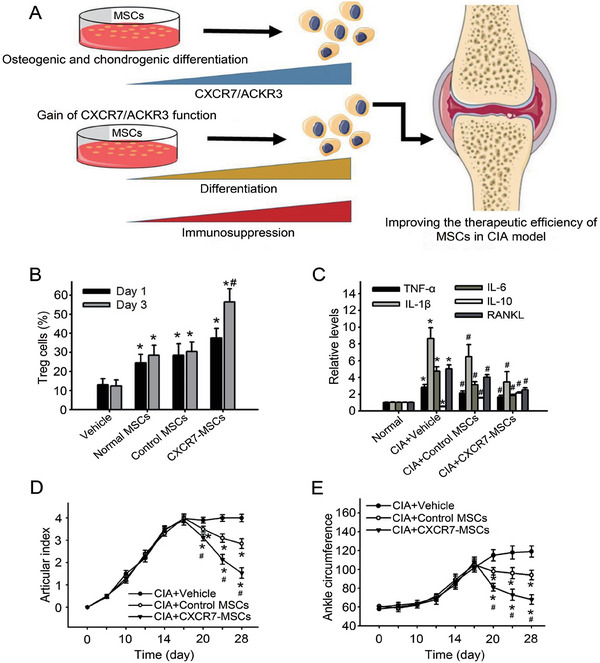
Mechanism and effect of MSCs with high expression of CXCR7 in treatment of arthritis. A) CXCR7‐MSCs in treatment of CIA model. B) Differentiation of Tregs cocultured with MSCs or CXCR7‐MSCs. Data are shown as mean ± SD (*n* = 9; ^*^ *p* < 0.001 compared to the normal group without CIA, ^#^ *p* < 0.001 compared to the CIA group without any treatment). C) Levels of cytokines in the synovial were derived from rats in different groups. Data are shown as mean ± SD (*n* = 6; ^*^ *p* < 0.001 compared to normal rats, ^#^ *p* < 0.001 compared to CIA rats). D,E) Articular index and ankle circumference in CIA treated with MSCs or CXCR7‐MSCs. Data are shown as mean ± SD (*n* = 6; ^*^ *p* < 0.001 compared to normal rats, ^#^ *p* < 0.001 compared with CIA rats). Reproduced under the terms of the CC‐BY license.^[^ ^90^ ^]^ Copyright 2021, the authors.

In addition to inhibiting TNF‐α, gene editing also enhances the immunomodulatory ability of MSCs. Kim et al. constructed MSCs epigenetically modified by DNA methyltransferase and histone deacetylase inhibitor and showed that modified MSCs up‐regulated IDO and IL‐10 levels and inhibited T cell proliferation.^[^ ^87^ ^]^ Cytotoxic T lymphocyte‐associated antigen 4 (CTLA4) is a protein receptor that inhibits T cell activation. CTLA4‐IgG is the fusion protein of CTLA4 and IgG1Fc segments, which blocks T cell activation and is effective in RA patients.^[^ ^88^ ^]^ Choi et al. generated human A‐MSCs highly expressing CTLA4‐IgG (CTLA4Ig‐A‐MSCs), demonstrating that they significantly increased the Treg/Th17 ratio in vitro. CTLA4Ig‐A‐MSCs decreased T‐bet and GATA binding protein 3 expression in the CIA mice splenocytes and increased the ratio of Treg and Th17 cells more significantly than wild A‐MSCs.^[^ ^89^ ^]^ Wei et al. treated CIA mice with MSCs highly expressing C‐X‐C chemokine receptor 7 (CXCR7) (**Figure** **5**A).^[^ ^90^ ^]^ The results showed that the induced MSCs promoted Treg proliferation and induced IL‐10 expression to promote immunosuppression (Figure 5B). In addition, levels of pro‐inflammatory cytokines were significantly reduced in the CIA rats treated with CXCR‐MSCs (Figure 5C). Moreover, after treatment with CXCR‐MSCs, the arthritis index and ankle circumference of CIA rats were significantly improved, and the efficacy was better than that of MSCs (Figure 5D,E). IL‐10 is an anti‐inflammatory cytokine secreted by monocytes, macrophages, B cells, and T cells, suppresses inflammatory responses in RA, and improves disease progression.^[^ ^91^ ^]^ BM‐MSCs Transfected with adenovirus highly express IL‐10 and are used to treat the CIA mice. Compared with the normal BM‐MSCs group, the spleen and thymus index of IL‐10‐BM‐MSCs group decreased significantly, while Foxp3 protein expression was lower. This indicated that Treg cells increased in the IL‐10‐BM‐MSCs group and inhibited the autoimmune response.^[^ ^92^ ^]^ Transgenic MSC exosomes also enhance immunomodulatory effects. MicroRNA‐146a (MiR‐146a) is an immunomodulator that inhibits nuclear factor κ‐B (NF‐κB) signaling and reduces inflammatory phenotypes. MiR‐146a transduced MSC‐EVs increased IL‐10, TGF‐β, and Foxp3 in the CIA mouse‐derived splenocytes. This indicated that the transduced MSC‐EVs increased Treg proportion and anti‐inflammatory cytokines secretion.^[^ ^93^ ^]^

MSCs promote anti‐inflammatory cytokine production by increasing immune cells, such as Th2, Treg, Breg, and M2 macrophages. Developing more strategies to increase the anti‐inflammatory and immunomodulatory ability of MSCs is the direction of future research.

## 3 Promotion of Tissue Regeneration

RA is a systemic polyarthritis that may cause progressive damage to the bones and cartilage of joints. A major feature of RA is bone erosion. Bone erosion occurs mainly in three forms: local bone erosion of the joint margins and subchondral bone, bone erosion around the inflamed joint, and osteoporosis.^[^ ^94^ ^]^ Bone erosion occurs early in the onset of RA, sometimes weeks after diagnosis. More than 10% of patients developed bone erosion eight weeks after onset, and 60% developed bone erosion one year after onset. Bone erosion in RA patients develops as the disease progresses, leading to joint damage and dysfunction.^[^ ^95^ ^]^ The tissue repair mechanisms mainly include two aspects. First, MSCs down‐regulate osteoclast generation and pro‐inflammatory cytokines through paracrine action, and inhibit bone and cartilage erosion in RA. Second, MSCs directly differentiate into osteoblasts and chondrocytes to replace damaged joint tissue.

### 3.1 Inhibition of Bone and Cartilage Destruction

Chronic inflammation of RA causes synovial hyperplasia, which invades adjacent bone and cartilage, causing bone destruction. Osteoclasts are the cells resulting in bone erosion in RA, and macrophage‐colony stimulating factor (M‐CSF) and receptor activator of nuclear factor κ‐B ligand (RANKL) are key ligands that promote osteoclast formation.^[^ ^96^ ^]^ Osteoprotegerin (OPG), a competitive inhibitor of osteoblast‐derived RANK, inhibits osteoclast formation and reduces bone erosion. The RANKL‐RANK‐OPG axis regulates bone homeostasis by regulating osteoclasts.^[^ ^94^ ^]^ Oshita et al. found that hMSCs inhibit osteoclast formation by producing OPG.^[^ ^97^ ^]^

During the pathogenesis of RA, many cytokines activate the RANK pathway, promoting osteoclast formation. For example, TNF promotes osteoclast differentiation by promoting RANK and T cell nuclear factor (NFATc1) expression in osteoclast precursor cells.^[^ ^98^ ^]^ TNF also promotes M‐CSF expression by bone marrow stromal cells to stimulate osteoclast formation.^[^ ^99^ ^]^ In addition, TNF also acts directly on osteoclast precursor cells and promotes their differentiation into osteoclasts.^[^ ^100^ ^]^ Besides, other cytokines also cause bone erosion in RA. In the RA synovium, IL‐1 is produced by peripheral blood monocytes and macrophages and acts synergistically with TNF during osteoclast differentiation.^[^ ^101^ ^]^ IL‐6 is produced by RA fibroblast‐like synoviocytes (FLSs) and macrophages and promotes osteoclast differentiation by stimulating FLS to express RANKL.^[^ ^102^ ^]^ IL‐17 is produced by Th17 cells and stimulates osteoclast differentiation by increasing RANK expression on osteoclast precursor cells.^[^ ^103^ ^]^ IL‐17 also induces osteoblasts to express PGE‐2, enhancing osteoclast differentiation and function.^[^ ^104^ ^]^ In addition to osteoclast activation, RA bone destruction is also associated with the inhibition of osteoblast differentiation and impaired function.^[^ ^105^ ^]^ TNF inhibits osteoblast differentiation by promoting the degradation of transcription factor Runx2 and induces osteoblast apoptosis.^[^ ^106^ ^]^ IL‐1 inhibits osteoblast proliferation and migration, leading to a decrease in bone erosion sites.^[^ ^107^ ^]^ Bone morphogenetic protein (BMP) promotes osteoblast differentiation, and IL‐6 inhibits this effect.^[^ ^108^ ^]^

MSCs inhibit pro‐inflammatory cytokine production, including TNF‐α and IL‐1β, critical mediators of articular cartilage destruction and bone erosion in RA.^[^ ^109^ ^]^ MSCs regulate immune cells, such as T cells and macrophages, and are involved in the pathogenesis of RA. MSCs inhibit the activation and proliferation of these cells, suppress the inflammatory response, and reduce damaging factors released that cause damage to articular cartilage and bone. In RA, the expression and activity of MMPs are increased, causing articular cartilage and bone destruction. MSCs secrete tissue inhibitors of matrix metalloproteinases to regulate matrix metalloproteinases (MMPs) and protect articular cartilage and bone tissue. MSCs directly inhibit MMP production and activation by synovial fibroblasts and immune cells. By reducing MMP levels, MSCs maintain articular cartilage and bone integrity.^[^ ^110^ ^]^

**Figure 6:**
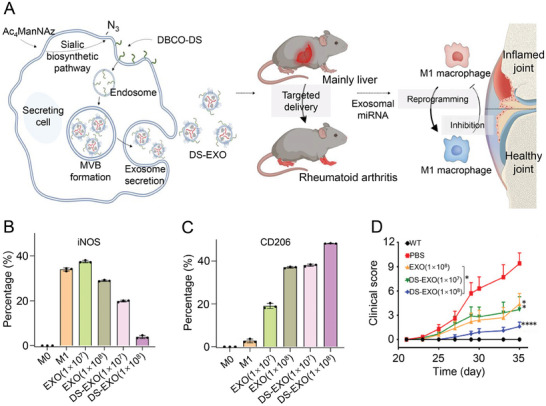
Mechanism and effect of MSC‐derived EXOs for treatment of CIA mice. A) MSC‐derived EXOs for CIA mice. B) Expression of iNOS in EXO‐treated RAW264.7 cells culture medium. Error bars represent SD (*n* = 3). C) Expression of CD206 in EXO‐treated RAW264.7 cells culture medium. Error bars represent SD (*n* = 3). D) Arthritis score of CIA mice after EXO treatment. Data are shown as mean ± SD (*n* = 3; ^*^ *p* < 0.05, ^**^ *p* < 0.01, ^***^ *p* < 0.001, ^****^ *p* < 0.001). Reproduced under the terms of the CC‐BY‐NC license.^[^ ^111^ ^]^ Copyright 2021, the authors.

In RA, M1 macrophages produce inflammatory cytokine, promoting osteoclast production and bone erosion. Therefore, inhibiting M1 and promoting M2 polarization are essential to reduce bone erosion in the RA therapy. You et al. performed metabolic engineering editing of A‐MSC‐derived exosomes (EXOs) to produce dextran sulfate EXOs (DS‐EXOs) targeting macrophages in the RA joints (**Figure** **6**A).^[^ ^111^ ^]^ After treatment with DS‐EXOs, inducible nitric oxide synthase (iNOS) expression in macrophages decreased, while CD206 expression increased significantly (Figure 6B,C), suggesting that exosomes promote the polarization of M1 into M2. Besides, the arthritis score of CIA mice decreased significantly after DS‐EXOs systemic administration (Figure 6D), indicating that DS‐EXOs reduce bone erosion and joint inflammation by altering macrophage phenotypes.

Interestingly, in RA patients, microRNAs (miRNAs) contribute to bone destruction by promoting osteoclast formation. For example, the increased expression of miRNA155 in fibroblasts and macrophages from RA patients results in an increase in the level of TNF, IL‐1, and IL‐6, leading to osteoclast infiltration.^[^ ^112^ ^]^ Blueml et al. found that osteoclast infiltration and joint destruction were significantly decreased in the CIA mice in which the miRNA155 gene was knocked out.^[^ ^113^ ^]^ Up‐regulation of miRNA‐233 in synovial cells and T cells of RA patients promoted osteoclast proliferation by inhibiting the macrophage colony‐stimulating factor receptor. The inhibition of miRNA‐233 expression in the CIA mice reduces osteoclast formation and bone destruction.^[^ ^114^ ^]^

Although many clinical drugs relieve the symptoms of RA, traditional drugs have a poor effect on repairing bone and cartilage. Because of their excellent proliferation and differentiation abilities, MSCs have been extensively studied in regenerative medicine. However, there are some limitations in the tissue regeneration capacity of MSCs, such as low survival rate, limited engraftment, and low homing and differentiation efficiency.^[^ ^90^ ^]^ The role of MSCs in the RA joint repair mainly includes the following two mechanisms. First, MSCs regulate the RANKL/OPG system through paracrine action and down‐regulate the generation of osteoclasts to inhibit bone and cartilage erosion in RA. Second, MSCs directly differentiate into osteoblasts and chondrocytes to replace damaged articular tissue, thus directly promoting joint repair of RA.^[^ ^115^ ^]^

The protective effects of MSCs on bone and cartilage in RA have been validated in vivo and in vitro. In the CIA rats, treatment with UC‐MSCs down‐regulated the expression of osteoclast‐inducing genes RANKL, RANK, and NFATc1 in the inflamed joints. In addition, MSCs inhibit MMPs and osteoclast‐specific genes tartrate resistant acid phosphatase expression. In conclusion, MSCs play an osteoprotective role in CIA by inhibiting the formation of osteoclasts.^[^ ^116^ ^]^ Zhou et al. studied the microstructure and mechanical behavior of the tibia of CIA mice. The results showed that bone erosion in the CIA mice was mainly manifested in the adjacent callus trabecular bone and metaphyseal cortical bone, and the mechanical strength of the proximal tibia was weakened. Gingival mesenchymal stem cell (GMSC) treatment alleviated bony erosion of the callus and trabecular bone and enhanced the mechanical properties of the proximal tibia.^[^ ^117^ ^]^ Gao et al. injected BM‐MSCs into CIA rats through the tail vein, and the results showed that joint inflammation and bone destruction were relieved. This is achieved by suppressing CXCL10/CXCR3 and modulating the RANKL/OPG ratio.^[^ ^118^ ^]^ A‐MSCs inhibited mouse and human osteoclastogenesis and NF‐κB P65/P50 levels, and CD39 inhibitors blocked this inhibition. The expression of P65/P50 and RANKL in synovial tissue was significantly reduced by injection of A‐MSCs before the onset of CIA mice. This suggests that the inhibition of RANKL and osteoclasts by A‐MSCs is achieved through the CD39 pathway.^[^ ^119^ ^]^ Luo et al. found that dental MSCs generated osteoclastogenesis and reduced bone erosion in vivo and in vitro. This effect could be attenuated by blocking CD39/CD73/adenosine receptors, suggesting that GMSCs inhibit RANKL‐mediated osteoclastogenesis through CD39/CD73/adenosine signaling.^[^ ^96^ ^]^

However, under the solid inflammatory microenvironments in the RA joints, both the survival and function of MSCs are affected.^[^ ^120^ ^]^ TNF‐α is a central inflammatory cytokine in RA. It suppresses the proliferation and function of MSCs by inducing autophagy/apoptosis and increases the risk of tumorigenesis.^[^ ^121^ ^]^ Therefore, inhibiting the effect of TNF‐α may be a way to enhance the tissue protective ability of MSCs. The TNFRII‐MSCs that Zhao et al. mentioned above have a stronger immunosuppressive effect and regulate RANKL/OPG levels in chondrocytes and FLS, showing a stronger chondroprotective effect. In addition, Zhao et al. evaluated the chondroprotective effect of TNFRII‐MSCs on CIA mice. After TNFRII‐MSC treatment, the expression of type II collagen (Col‐II) was higher, and MMP‐13 expression was lower, suggesting that TNFRII‐MSCs protect cartilage by regulating the metabolic balance of the cartilage matrix.^[^ ^42^ ^]^ Macrophages are the main cell line that secretes TNF‐α, and macrophages differentiate into osteoclasts after treatment with RANKL. Park et al. prepared TNFRII MSCs that secreted TNF‐α inhibitors by transfecting MSCs with micro‐circles. They found that monocyte‐macrophages treated with supernatant of TNFRII MSCs had reduced ability to secrete TNF‐α and differentiate into osteoclasts.^[^ ^43^ ^]^

FLSs are one of the primary cells in the synovial intima structure. They play a crucial role in RA pathogenesis by promoting synovial inflammation and joint destruction. FLSs contribute to RA joint destruction through the overproduction of extracellular matrix and MMP. Studies have shown that MSCs inhibit RA synovial fibroblast activation, thus protecting the joint. The activated FLSs express RANKL to promote the differentiation of macrophages into osteoclasts. MMPs secreted by FLS directly destroy bone and cartilage.^[^ ^122^ ^]^ Therefore, inhibiting the proliferative function of FLSs reduces the generation of osteoclasts and MMPs to protect bone and cartilage. Liu et al. transplanted two kinds of scaffolds, including fibrin gel and hydrogel, with MSCs into subchondral defects of AIA mice, and they observed that both scaffolds loaded MSCs reduced FLS proliferation and infiltration of inflammatory mediators, reducing cartilage destruction.^[^ ^123^ ^]^ In addition, MSCs‐derived extracellular vesicles with high expression of specific miRNAs also inhibit FLS proliferation and migration and play a protective role on bone and cartilage.

MiRNAs are small non‐coding RNAs that mediate gene expression post‐transcriptionally to degrade miRNA and inhibit translation.^[^ ^124^ ^]^ For example, MSC‐EVs promoted the proliferation of FLS in the CIA mice by transferring miR‐21 and inhibited the secretion of inflammatory factors. The effect of miR‐21 is achieved by inhibiting Krvppel‐like factor 4  gene expression, a transcription factor class regulating inflammatory responses.^[^ ^125^ ^]^ Meng et al. constructed MSCs‐EVs highly expressing miR‐124A (MSC‐124A‐EVs) and studied their effects on FLS cells in vitro. MSC‐124A‐EVs inhibited synovial cell proliferation and migration and promoted synovial cell apoptosis.^[^ ^126^ ^]^ MiR‐320 and CXCL9 are mutually expressed in synovial tissues from RA patients, and CXCL9 is the target of miR‐320. After coculture with FLS from RA patients, MSC‐EVs containing miR‐320 suppressed FLS activation, migration, and invasion by down‐regulating CXCL9. MSC‐EVs containing miR‐320 reduced bone and joint injury and arthritis progression in the CIA mice.^[^ ^127^ ^]^

**Figure 7:**
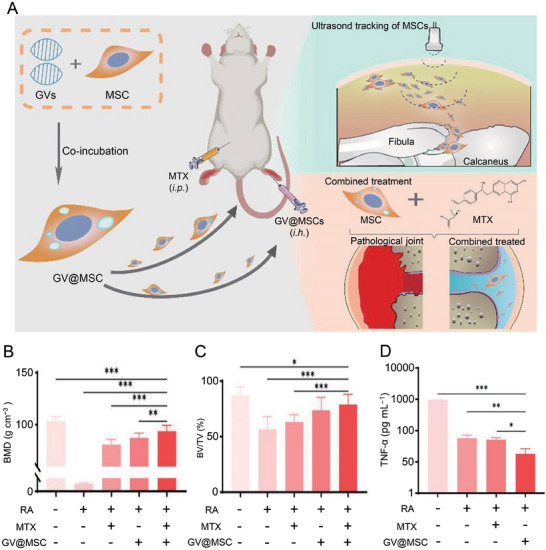
Mechanism and effect of GV@MSCs in RA therapy. A) GV@MSCs for real‐time imaging in RA therapy. B,C) BMD and BV/TV of different treatment groups were calculated using CT images. D) TNF‐α levels in serum of different groups. Data are shown as mean ± SD (*n* = 6; ^*^ *p* < 0.05, ^**^ *p* < 0.01, ^***^ *p* < 0.001). Reproduced under the terms of the CC‐BY license.^[^ ^130^ ^]^ Copyright 2022, the authors.

Although MSCs are essential in RA tissue protection and regeneration, the lack of real‐time imaging and tracking of MSCs makes the specific mechanisms unclear. Optical imaging time and spatial resolution are good, but the penetration depth and radiation intensity are limited. Magnetic resonance imaging has good tissue penetration strength but limited spatiotemporal resolution. In a study of brain injury repair, Li et al. labeled MSCs with an improved Prussian blue dye to give excellent photoacoustic contrast of MSCs. After the labeled MSCs are injected into the body, they can be tracked by rapid photoacoustic tomography with optical molecular probes.^[^ ^128^ ^]^ In addition, photoacoustic imaging technology also plays a role in tumor visualization.^[^ ^129^ ^]^ This tracking technique may be used in MSC therapy for RA. Gong et al. loaded gas vesicles (GVs) into MSCs for the treatment of RA and ultrasound imaging of MSCs (**Figure** **7**A).^[^ ^130^ ^]^ Ultrasound imaging has good tissue penetration depth and time‐space sensitivity. GVs‐loaded MSCs (GVs@MSCs) showed an excellent ultrasonic signal in the RA joint. GVs@MSCs combined with MTX were effective in the treatment of CIA rats. Bone mineral density (BMD) and bone volume over total volume (BV/TV) in the combined treatment group were higher than those treated with MTX and MSCs alone (Figure 7B,C). This therapeutic effect was associated with decreased TNF‐α content in the synovium of CIA rats (Figure 7D).

In short, the core of inhibiting bone and cartilage destruction is to reduce osteoclasts and FLS infiltration. In the future, more strategies to enhance the tissue protection role of MSCs in RA need to be developed.

### 3.2 Promotion of Bone and Cartilage Regeneration

MSCs differentiate into chondrocytes, specialized cells responsible for cartilage formation and maintenance. When introduced into the RA joint microenvironments, MSCs perform cartilage differentiation to relieve the destruction of RA articular cartilage.^[^ ^118^ ^]^ This differentiation process involves upregulating chondro‐specific genes and producing extracellular matrix components, such as Col‐II and proteoglycans, which are essential for cartilage structure and function.^[^ ^131^ ^]^ MSCs secrete various bioactive molecules, including growth factors, cytokines, and chemokines, which profoundly impact surrounding microenvironments. These secretory factors stimulate the proliferation and migration of endogenous chondrocytes, enhance extracellular matrix synthesis, and promote angiogenesis in the joints, all of which contribute to cartilage regeneration.^[^ ^118^ ^]^

MSCs also differentiate into osteoblasts, which promote bone formation. In RA, bone erosion and osteoporosis are common manifestations of an imbalance between bone resorption and formation.^[^ ^94^ ^]^ When exposed to osteogenic differentiation factors, MSCs differentiate into osteoblasts and contribute to the formation of new bone. This process involves the production of osteogenic markers, such as alkaline phosphatase and osteocalcin, as well as the deposition of a mineralized matrix to repair damaged bone.^[^ ^132^ ^]^ Like their role in cartilage regeneration, MSCs play a paracrine role in regulating bone remodeling in RA. They secrete several factors, including BMP, insulin‐like growth factor‐1, and vascular endothelial growth factor, involved in bone formation and angiogenesis. These factors stimulate the proliferation and differentiation of osteoblasts, enhance bone matrix mineralization, and promote the recruitment of endothelial cells, thereby improving bone healing and remodeling.^[^ ^133^ ^]^

Although the profound value of MSCs in regenerative medicine has attracted wide attention, applying their repair capability in RA still faces significant challenges. For example, the regenerative effect of MSCs will be affected by the tissue source of MSCs and the route of administration. MSCs can be isolated and purified from various tissues, and it has been found that MSCs from different sources differ in their differentiation potential. Studies have shown that BM‐MSCs have better osteogenic and chondrogenic differentiation potential than A‐MSCs.^[^ ^134^ ^]^ In addition, synovium MSCs were shown to have a more robust chondrogenic capacity than BM‐MSCs, A‐MSCs, and periosteal MSCs in vitro. However, the osteogenic potential of synovium MSCs is lower than that of periosteal MSCs in vivo.^[^ ^135^ ^]^

Additionally, one of the advantages of MSCs in therapy is their ability to home to damaged tissues. Under the induction of chemokines, MSCs migrate to the injury site through adhesion, activation, entrapment, diapedesis, and migration.^[^ ^136^ ^]^ However, the expression of homing molecules is reduced in cells cultured for a long time, which reduces the homing efficiency of MSCs after systemic administration.^[^ ^137^ ^]^ Therefore, genetically, using virus transfection and other methods to modify MSCs to overexpress homing molecules like CXCRs may be a feasible method to improve the homing ability of cells. This technique introduces a pre‐constructed gene cassette into a viral vector, which is then used to infect MSCs. In MSCs, viral vectors induce overexpression of specific genes affecting homing factors.^[^ ^138^ ^]^ CXCR7 is an essential gene responsible for MSC adhesion and survival, and its overexpression promotes the migratory potential of MSCs.^[^ ^139^ ^]^ Wei et al. found that MSCs overexpressing CXCR7 promoted osteogenic and chondrogenic differentiation. Further research found that this promotion is achieved through several pathways, such as peroxisome proliferators‐activated receptors, Hedgehog, and Notch.^[^ ^90^ ^]^

Since RA is a systemic disease, systemic administration of MSCs is the primary method of administration in RA animals. Systemic administration is convenient and rapid but has some disadvantages. For example, MSCs were significantly diluted in the blood after intravenous administration and remained in the pulmonary vessels, resulting in fewer cells reaching the treatment site.^[^ ^120^ ^]^ Several techniques have been developed to overcome the low efficiency of systemic administration of MSCs to improve their homing efficiency. For instance, anticoagulants or vasodilators reduce the pulmonary retention of cells and increase homing efficiency.^[^ ^140^ ^]^

**Figure 8:**
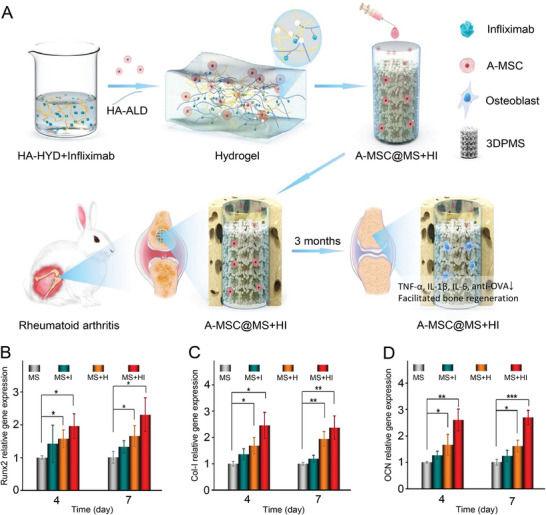
Mechanism and effect of A‐MSCs and infliximab hydrogel loaded with 3D metal scaffolds in RA therapy. A) A‐MSCs loaded with infliximab hydrogel in RA therapy. B) Runx2 gene expression of A‐MSCs cultured on different 3D metal scaffolds. C) Col‐I gene expression of A‐MSCs cultured on different 3D metal scaffolds. D) *OCN* gene expression of A‐MSCs cultured on different 3D metal scaffolds. Data are shown as mean ± SD (*n* = 3; ^*^ *p* < 0.05, ^**^ *p* < 0.01, ^***^ *p* < 0.001). Reproduced with permission.^[^ ^142^ ^]^ Copyright 2021, Elsevier.

Alternatively, the direct transplantation of MSCs into damaged joint sites may be a logical approach to improve the efficiency of cell delivery. Directly administered cells allow for easier homing and less dilution and loss of cells and active factors in the blood.^[^ ^120^ ^]^ For example, in bone tissue engineering, intra‐articular administration can be used for osteoarthritis and cartilage repair.^[^ ^141^ ^]^ Therefore, although RA is a systemic disease, local administration can also be used under specific strategies to ensure cell survival and delivery. For example, under the condition of RA with severe joint damage, the transplantation of specific scaffold‐loaded MSCs into the joint defect shows a better joint repair effect. Liu et al. combined hydrogel‐loaded MSCs with microfracture to treat cartilage defects in the CIA mice. Synovial swelling in the experimental group was considerably relieved, and cartilage damage was significantly reduced.^[^ ^33^ ^]^ Zhao et al. designed a three‐dimensional (3D) metal scaffold loaded with infliximab hydrogel and demonstrated its ability to increase A‐MSCs survival, proliferation, and osteogenic differentiation in vivo and in vitro (**Figure** **8**A).^[^ ^142^ ^]^ This may be because infliximab inhibits TNF‐α, reducing the adverse effects of the inflammatory milieu. Several scaffolds equipped with A‐MSCs in RA animal models promoted Runx2, osteocalcin, and Col‐I gene expression to varying degrees (Figure 8B–E). This indicates that in the RA animal model with severe cartilage damage, local MSCs transplantation significantly reduced cartilage damage and improved the repair effect.

**Figure 9:**
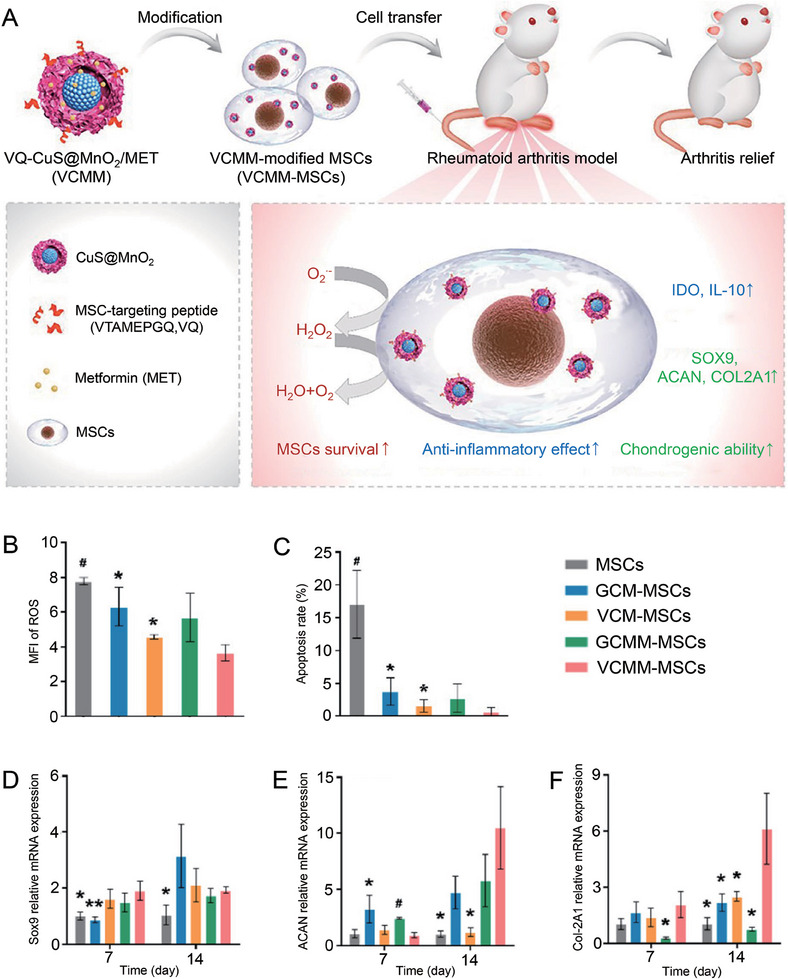
Mechanism and effect of VQ‐CuS@MnO_2_/MET(VCMM) nanoparticles modified MSCs (VCMM‐MSCs) in RA therapy. A) VCMM‐MSCs for RA therapy. B) The mean fluorescence intensity (MFI) of ROS was measured from five random locations of three independent samples. C) Cell apoptosis rate of different MSCs in the presence of H_2_O_2_. Data are shown as mean ± SD (*n* = 3; ^*^ *p* < 0.05, ^#^ *p* < 0.001 compared to VCMM‐MSC group). D–F) Relative mRNA expression of chondrogenic genes in MSCs after chondrogenic incubation for 7 and 14 days. Data are shown as mean ± SD (*n* = 3; ^*^ *p* < 0.05, ^**^ *p* < 0.01, ^#^ *p* < 0.001, ^##^ *p* < 0.0001 compared to VCMM‐MSC group). Reproduced with permission.^[^ ^143^ ^]^ Copyright 2022, Elsevier.

Interestingly, in addition to tissue sources and drug administration routes, the tissue repair capability of MSCs is affected by inflammatory microenvironments. In the RA‐inflamed joints, survival and chondrogenic differentiation of MSCs are inhibited. Because of multi‐directional differentiation ability, uncontrolled differentiation of MSCs is associated with the risk of tumorigenesis. Therefore, reducing the influence of inflammatory microenvironments on MSCs and promoting osteogenic and chondrogenic differentiation of MSCs is essential. Lu et al. developed a nanoparticle (VQ‐CuS@MnO_2_/MET) composed of CuS, MnO_2_, metformin (MET), and MSCs targeting peptide (VQ) for RA therapy (**Figure** **9**A).^[^ ^143^ ^]^ Cu and Mn are involved in superoxide dismutase (SOD) synthesis and have anti‐inflammatory effects. In addition, Cu and Mn promoted the chondrogenic differentiation of MSCs. MET enhanced the anti‐inflammatory ability of MSCs. In the RA inflammatory microenvironments simulated by hydrogen peroxide (H_2_O_2_), intracellular ROS of MSCs modified by nanoparticles decreased significantly (Figure 9B). Besides, the apoptosis rate of MSCs modified by nanoparticles was significantly reduced (Figure 9C). The expression of chondrogenic genes in MSCs modified by nanoparticles significantly increased than that of the unmodified MSCs (Figure 9D–F). In short, MSCs modified by nanoparticles decreased apoptosis rate by inhibiting oxidative microenvironments, thus increasing chondrogenic differentiation. Another study also increased the regeneration capacity of MSCs by reducing the effects of inflammatory microenvironments. Zhao et al. developed a nano‐enzyme‐enhanced hydrogel as a carrier of MSCs for RA therapy. The hydrogel decomposed ROS, which promoted the survival, proliferation, and osteogenic differentiation of MSCs in vitro. The in vivo effect was verified by the RA rabbit model induced by ovalbumin (OVA). The MSCs‐loaded hydrogel was injected into a 3D‐printed titanium alloy scaffold and implanted into the distal femur. The two control groups were titanium alloy stents with or without MSCs. Compared with the control group, the nano‐enzyme hydrogel loaded with MSCs significantly enhanced the effect of bone regeneration and bone integration.^[^ ^144^ ^]^

To improve the differentiation capacity of MSCs in RA, some studies have also achieved favorable results by coculturing MSCs with cells and cytokines. Growth differentiation factor 5 (GDF‐5) belongs to the family of BMPs and TGF‐β produced by FLS and articular chondrocytes, demonstrating the roles in promoting cartilage differentiation and repair in vivo and in vitro.^[^ ^145^ ^]^ A study cocultured UC‐FLS, which significantly increased GDF‐5 protein production and chondrogenic differentiation, suggesting that the coculture of FLS with MSCs may increase chondrogenesis via GDF‐5.^[^ ^146^ ^]^ IL‐37 is an anti‐inflammatory cytokine that inhibits the expression of inflammatory factors, such as TNF‐α, IL‐6, and IL‐17, in PBMCs of RA patients.^[^ ^147^ ^]^ IL‐37 treatment significantly increased the expression of osteoblast‐specific genes, mineral deposition, and alkaline phosphatase activity in MSCs. This IL‐37 effect was partially inhibited by adding phosphatidylinositol 3‐kinase (PI3K) inhibitors, indicating that IL‐37 increases osteogenesis by activating PI3K signal.^[^ ^148^ ^]^

IL‐6 activates RANKL in FLS in the inflammatory microenvironments to promote osteoclast formation, resulting in bone erosion in RA patients.^[^ ^149^ ^]^ In addition, IL‐6 increases MSC proliferation by promoting SATA3 phosphorylation. Since MSCs do not express IL‐6 receptor (IL6R), some researchers have combined IL‐6 and soluble IL6R (sIL6R) in A‐MSCs to promote osteogenic differentiation. This process may be partially accomplished through the SATA3‐Osterix pathway.^[^ ^150^ ^]^ On this basis, Yamagata et al. developed an sIL‐6R‐based system to promote RA cartilage regeneration by implanting sIL‐6R‐pretreated MSCs onto poly membranes and into AIA rats, which resulted in enhanced articular cartilage repair.^[^ ^151^ ^]^

In summary, MSCs promote bone and cartilage regeneration in the RA‐inflamed joints by differentiating into osteoblasts and chondroblasts. How to reduce the influence of inflammatory microenvironments on MSCs and promote the differentiation of MSCs into bone and cartilage is the primary research direction in the future.

## 4 Challenges and Perspectives

RA therapy remains a significant challenge. Despite the availability of several conventional drugs, their therapeutic efficacy is limited and often accompanied by a range of side effects like the liver and kidney function impairment and infection risk.^[^ ^152^ ^]^ MSCs have shown promising results in RA preclinical and small‐scale clinical trials over the past few years.^[^ ^153^ ^]^ The following are representative clinical trials of MSCs in the treatment of RA over the past decade (**Table** **2**). These trials explored the different sources, doses, and patient populations of MSCs. Many trials mainly focused on evaluating the safety and feasibility of MSCs in the treatment of RA. Among these clinical trials, UC‐MSCs were the most frequently used. Wang et al. conducted two studies. One study included 172 patients with RA.^[^ ^154^ ^]^ Patients received DMARDs in combination with allogeneic UC‐MSCs (2.0 × 10^7^/patient), with DMARDs alone as a control. No serious adverse reactions were observed in the eight months after treatment. The clinical scores and serum inflammatory factor levels of the combined treatment group were better than those of the control group. Another study included 64 RA patients observed for three years after the same treatment. Similarly, the patient's condition was significantly improved, and there were no serious adverse reactions.^[^ ^155^ ^]^ In another study, Park et al. divided 9 RA patients into three groups and received an intravenous infusion of different concentrations of UC‐MSCs. Patients in three groups received an intravenous infusion of 2.5 × 10^7^, 5.0 × 10^7^, or 1.0 × 10^8^ UC‐MSCs, respectively. There was no significant toxic reaction after treatment. In addition, the subjects' serum inflammatory factors and joint disease activity scores decreased. It should be noted that the treatment effect was better when the concentration of MSCs was higher.^[^ ^156^ ^]^

**Table 2:** Summary of MSCs‐involved Clinical studies for RA therapy.

Source of MSCs	Enrollment	Treatment	Phase	Reference
UC‐MSCs	172 patients	DMARDs with/without 4.0 × 10^7^ cells/patient via intravenous injection	Phase I/II	[154]
UC‐MSCs	64 patients	2.0 × 10^7^ cells/patient via intravenous injection	Phase I/II	[155]
UC‐MSCs	9 patients	2.5 × 10^7^, 5.0 × 10^7^ or 1.0 × 10^8^ cells/patient via intravenous infusion	Phase I	[156]
UC‐MSCs	105 patients	1.0 × 10^6^ cells/kg body weight (BW) via intravenous infusion	Phase I/II	[157]
UC‐MSCs	63 patients	1.0 × 10^6^ cells/(kg BW) via intravenous infusion with/without intramuscular infusion of IFN‐γ	Phase I/II	[39]
BM‐MSCs	9 patients	1.0 × 10^6^ cells/(kg BW) via intravenous infusion	Phase I	[158]
BM‐MSCs	30 patients	42 ± 4.0 × 10^6^ cells intra‐knee injection	Phase I/II	[159]
A‐MSCs	53 patients	1, 2, or 4.0 × 10^6^ cells/(kgBW) via intravenous infusion three times	Phase I/II	[160]
A‐MSCs	15 patients	2.0 × 10^8^ cells/patient via intravenous infusion	Phase I/II	[161]

Although MSCs have achieved good results in the above studies, they have not been fully effective in other studies. Yang et al. selected 105 refractory RA patients and randomly divided them into a treatment group and a control group. 52 patients were treated with 1.0 × 10^6^ UC‐MSCs/kg intravenously. After 12 weeks of treatment, only 28 patients had a good response, and the rest had no clinical response. Interestingly, elevated serum IFN‐γ was observed in patients in the response group.^[^ ^157^ ^]^ In another study, 63 patients with refractory RA were treated with UC‐MSCs with or without IFN‐γ. Interestingly, patients in the MSCs plus IFN‐γ treatment group had a much higher response rate than those in the no‐combination group.^[^ ^39^ ^]^ In summary, these studies once again suggest that there may be a “permission” relationship between the therapeutic effect of MSCs and some inflammatory factors. In addition, the different therapeutic effects shown by MSCs may be related to differences between patients.

In addition to an umbilical cord, MSCs also come from bone marrow. In one study, 9 refractory RA patients received 1.0 × 10^6^ BM‐MSCs/kg intravenous infusion. After a 12‐month follow‐up, the clinical score of patients decreased significantly, and Treg/Th17 proportion increased.^[^ ^158^ ^]^ In another study, 30 RA patients were randomly assigned to a treatment group or a placebo group. BM‐MSCs were injected into the knee of 15 RA patients. After a 12‐month follow‐up, no adverse reactions were found. In addition, the clinical manifestations of the patients have been improved.^[^ ^159^ ^]^ In a word, both intravenous and local injection of MSCs achieve good clinical results. However, RA is a systemic disease involving multiple joints, and systemic injection is more beneficial for MSCs to reach the affected joints. Therefore, intravenous injection is more common than local injection in clinical trials.

In the first randomized, placebo‐controlled clinical trial of A‐MSC for RA, 53 refractory RA patients were treated. The treatment group received three intravenous transfusions of A‐MSCs over 15 days at doses of 1, 2, and 4.0 × 10^6^ cells (kg BW)^−1^. After 6‐month follow‐up, the clinical effect of treatment group was obvious. Although adverse events, such as infection and fever, occurred in the treatment group, these reactions were considered to have nothing to do with treatment.^[^ ^160^ ^]^ Another clinical trial further confirmed the safety and efficacy of A‐MSCs in RA therapy. Fifteen active RA patients were enrolled in the study. The subjects received a single intravenous injection of 2.0 × 10^8^ A‐MSCs and received long‐term follow‐up for 52 weeks. After treatment, the patient's clinical scores and inflammatory indicators were significantly improved, and inflammatory index was significantly improved. The hematology, liver, and kidney function were normal. In addition, no serious adverse events occurred.^[^ ^161^ ^]^

Overall, these studies reported that the MSC therapy for RA appeared to be safe, with few significant adverse effects. The MSC infusion was usually well tolerated by patients. Although promising signs of efficacy had emerged, the results were usually variable in terms of significant clinical improvements in RA. After treatment with MSCs, the disease activity score was improved, inflammation markers decreased, and the joint function was enhanced. However, these effects varied across trials and patients. In addition, determining the optimal dose and route of administration of MSCs in RA remains an ongoing challenge. Different doses and routes were used to deliver MSCs, but the most effective method has not been determined. The study used MSCs from different sources, such as bone marrow, adipose tissue, and umbilical cord. The characteristics and efficacy of MSCs from different sources may vary, and the choice of MSC source may affect the treatment outcome. Different sources allow for flexibility in treatment strategies, but optimizing protocols and understanding the specific benefits and limitations of each source remains critical to advancing MSCs‐based RA treatments. The researchers also explored MSC therapy in combination with other treatments, such as DMARDs, to assess synergies and potentially improve treatment outcomes. Although the combination of MSCs with existing RA treatments is theoretically promising, more reliable clinical evidence is essential to verify the effectiveness, safety, and long‐term outcomes of these combinations. More high‐quality research is needed to determine the most effective and safe combination of options. In addition, patient‐specific factors, such as disease duration, severity, and individual immune response, may influence response to MSC therapy. Understanding these factors is critical to identifying the best candidates for RA therapy.

In addition to the above problems exposed in clinical trials, preclinical trials also provide valuable experience for the clinical transformation of MSCs. There are some adverse factors in the efficacy of MSCs in RA preclinical trials. The immunosuppression potential of MSCs can be affected by cytokines, such as chemokines and growth factors, in the microenvironments. For example, the presence of IFN‐γ activates the immunosuppressive ability of MSCs. On the contrary, TNF‐α induces the apoptosis of MSCs, thus affecting the therapeutic effect.^[^ ^34^ ^]^ Besides, the ability of MSCs to regulate inflammation also depends on their ability to home to the inflammatory site. Stem cell homing is a complex process. The homing properties of MSCs can be modified and influenced by pretreatment and cell engineering to create a better home for specific tissues, thereby enhancing their therapeutic effect. In addition to the immunomodulatory capability, MSCs possess excellent tissue repair capability. This means that MSCs not only inhibit the inflammation of RA joints by regulating immune cells but also effectively prevent and treat bone erosion in RA. However, in the inflammatory microenvironments of the joints of RA patients, MSCs are affected by the inflammatory microenvironments and impair their tissue repair ability.^[^ ^120^ ^]^ Therefore, reducing the influence of MSCs in inflammatory microenvironments is feasible by inhibiting inflammatory factors like TNF‐α. In addition, the direct transplantation of MSCs into inflamed joint sites through bioactive carriers also effectively reduces the wastage of MSCs and enhances their bone repair capacity.

In view of the present situation and experience of above clinical research and preclinical research, the following problems still need to be solved in the clinical transformation of MSCs. First, there is a lack of standardized protocols for MSC treatment of RA, including MSC production and treatment protocols. The source, dose, treatment regimen, and duration of MSCs vary in different studies. To apply MSCs in RA therapy on a large scale, it is necessary to produce and prepare MSCs in large quantities. Therefore, it is necessary to establish standardized MSC production and preparation protocols and carry out strict quality control and safety evaluations to ensure the quality and stability of MSCs. In addition to the production plan, standardized treatment plans must also be established. The source, dosage, application route, duration, and course of treatment of MSCs must be clarified and standardized. For example, should MSCs be preconditioned in vitro before being used in human RA? Is there an optimal treatment time for MSCs to treat RA? Should MSCs be used alone or in combination with other therapies? How can the depletion of MSCs during homing and in the inflammatory microenvironments be reduced? These questions need accurate and standardized answers.

However, the efficacy and safety of MSC therapy are uncertain. Although small‐scale clinical trials have shown efficacy, the degrees of efficacy, duration, and safety have not been fully confirmed. Therefore, conducting more extensive randomized controlled clinical trials of MSCs in RA therapy is necessary. In addition, long‐term follow‐up studies should be strengthened, including long‐term immunological surveillance and the risk of infection and tumor. In time, a safety monitoring system should be established to deal with possible side effects. Patient screening is a challenge in large‐scale clinical practice. Patient screening is essential because their age, disease severity, medical history, and comorbidities affect the efficacy of MSCs. Strict patient inclusion and exclusion criteria for using MSCs in RA should be developed, and the collection and analysis of clinical data should be strengthened to evaluate the efficacy and safety of MSCs for RA.

In general, in preclinical studies, more approaches should be explored to improve the anti‐inflammatory and tissue repair potential of MSCs in RA therapy. Clinical application and promotion of MSCs in RA therapy require addressing its difficulties and challenges through standardized treatment regimens, large‐scale clinical studies, and safety monitoring.

## 5 Conclusion

In conclusion, the MSCs‐involved strategy is a promising treatment modality for RA due to its excellent anti‐inflammatory and tissue repair ability. By exploring the interaction mechanism between MSCs and RA inflammatory microenvironments, the researchers will develop more strategies to improve the function of MSCs. Although there are still many problems to be solved in the large‐scale clinical application and promotion of MSCs, MSCs will exert a more essential role in the clinical treatment of RA in the foreseeable future.

## Conflict of Interest

The authors declare no conflict of interest.
